# Asymptomatic Medullary Thyroid Carcinoma With Isolated Carcinoembryonic Antigen Elevation in a Patient With Chronic Kidney Disease: A Case Report

**DOI:** 10.1002/ccr3.73580

**Published:** 2026-09-21

**Authors:** Orhan Altınyüzük, Bartu Ediz, Serkan Feyyaz Yalın, Ergün Parmaksız

**Affiliations:** ^1^ Department of Internal Medicine Kartal Dr. Lütfi Kırdar Training and Research Hospital, University of Health Sciences Istanbul Turkey; ^2^ Department of Nephrology Kartal Dr. Lütfi Kırdar Training and Research Hospital, University of Health Sciences Istanbul Turkey

**Keywords:** calcitonin, carcinoembryonic antigen, chronic kidney disease, medullary thyroid carcinoma, procalcitonin

## Abstract

Medullary thyroid carcinoma (MTC) is rare and typically presents with a palpable nodule. We report a 64‐year‐old asymptomatic woman with chronic kidney disease (CKD) diagnosed with MTC after incidental procalcitonin elevation on routine labs, highlighting procalcitonin as an atypical clue and the need to distinguish tumor‐related from CKD‐related hypercalcitoninemia.

## Key Clinical Message

Isolated procalcitonin elevation—a biomarker not conventionally linked to thyroid disease—may unmask asymptomatic medullary thyroid carcinoma even without a palpable nodule. In chronic kidney disease, distinguishing tumor‐related hypercalcitoninemia from renal‐related elevation is essential, as this distinction directly changes diagnostic and therapeutic decisions.

## 1 Introduction

Medullary thyroid carcinoma (MTC) is a rare malignancy that arises from the parafollicular C cells of the thyroid gland and, owing to its neural crest origin, is classified among the neuroendocrine tumors. Although it accounts for only 1%–2% of all thyroid cancers, MTC is responsible for a disproportionately large share of thyroid cancer–related deaths, a disparity that underlies the clinical rationale for diagnostic and therapeutic rigor in this disease [1, 2].

Approximately one‐quarter of cases arise in the setting of autosomal dominantly inherited MEN type 2A, MEN type 3, and familial MTC syndromes, which result from germline mutations in the RET proto‐oncogene, while the large majority of cases are sporadic. A somatic RET mutation is identified in roughly 60% of sporadic cases, and tumors harboring the most common of these, the RET p.Met918Thr alteration, follow a markedly more aggressive course; a substantial proportion of RET‐negative cases instead carry RAS‐family mutations. Because the specific codon affected by the RET mutation determines both the tumor's aggressiveness and the risk of concurrent pheochromocytoma and hyperparathyroidism, current guidelines have adopted a risk classification based on genotype–phenotype correlation; as family history may be absent in up to half of MEN2 cases, germline RET screening is recommended even in apparently sporadic patients [1, 2].

Although MTC most commonly presents clinically as a palpable thyroid nodule, a substantial proportion of patients present at diagnosis with cervical or distant metastasis, and occasionally with secretory manifestations such as diarrhea or ectopic Cushing syndrome resulting from calcitonin excess. Calcitonin and carcinoembryonic antigen (CEA) remain the cornerstones of diagnosis and follow‐up, and because the preoperative diagnostic accuracy of fine‐needle aspiration cytology remains limited, at 56%–65%, complementary biomarkers such as procalcitonin have been shown to reduce diagnostic uncertainty, particularly in indeterminate cases. Furthermore, calcitonin and CEA doubling times are considered an even stronger predictor of disease course than clinical staging [2, 3].

Given this heterogeneous clinical course, the associated diagnostic challenges, and the rapidly evolving landscape of RET‐targeted therapies, each additional MTC case reported in the literature carries individual value. Here, we present a case of MTC that manifested through an isolated procalcitonin elevation incidentally detected during routine testing in an asymptomatic patient, and that posed a diagnostic challenge in the setting of underlying chronic kidney disease, discussed in light of the current literature.

## 2 Case History and Examination

A 64‐year‐old woman with a known history of diabetes mellitus, hypertension, and chronic kidney disease was referred for further evaluation after an elevated procalcitonin level was incidentally identified on routine laboratory testing. She was entirely asymptomatic, and there was no family history of thyroid cancer or other endocrine neoplasia. Physical examination revealed no palpable thyroid nodule or cervical lymphadenopathy, and no stigmata suggestive of MEN2B, such as a marfanoid habitus or mucosal neuromas, were observed.

## 3 Methods

Given the incidental procalcitonin elevation together with thyroid nodularity on subsequent imaging, the differential diagnosis included medullary thyroid carcinoma, C‐cell hyperplasia, and other calcitonin‐secreting neuroendocrine neoplasms; renal impairment as an isolated cause of hypercalcitoninemia was also considered given the patient's chronic kidney disease.

Neck ultrasonography demonstrated solid, hypoechoic nodules measuring 21 × 14 mm in the right lobe and 15 × 13 mm in the left lobe. Laboratory evaluation revealed a serum calcitonin level of 2100 ng/L and a CEA level of 20.8 ng/mL; plasma free metanephrine and normetanephrine levels were within normal limits, serum calcium was 9 mg/dL, and PTH was 122 pg/mL. Germline RET mutation analysis could not be performed.

Fine‐needle aspiration biopsy of the left lobe nodule was reported as consistent with medullary thyroid carcinoma; calcitonin was positive in the washout fluid, and immunocytochemistry showed positivity for chromogranin A and CEA (monoclonal), negativity for thyroglobulin, focal positivity for PAX8, and focal, weak positivity for TTF‐1. Computed tomography of the neck, thorax, and abdomen showed no evidence of distant metastasis. Staging 68Ga‐DOTATATE PET/CT demonstrated a 2 × 1.5 cm hypodense nodule in the left lobe, along with several lymph nodes showing tracer uptake in the cervical and bilateral axillary stations, more prominent on the left.

The patient underwent total thyroidectomy with bilateral central (including pretracheal) neck dissection.

## 4 Conclusions and Results

Pathological examination revealed a 2 cm, partially encapsulated, low‐grade medullary thyroid carcinoma in the left lobe without vascular or perineural invasion and with negative surgical margins; no C‐cell hyperplasia was identified, mitotic activity was 1/2 mm^2^, and the Ki‐67 proliferation index was 1%. Amyloid deposition could not be demonstrated by either histochemical (Congo red) or immunohistochemical staining. An incidental, 0.05 cm classic‐type papillary microcarcinoma was additionally identified in the right lobe. All 17 dissected lymph nodes (from the central, pretracheal, and right central compartments) showed reactive lymphoid hyperplasia, with no evidence of metastasis (pN0). Immunohistochemistry of the tumor cells demonstrated positivity for calcitonin, CEA, chromogranin A, synaptophysin, CK19, HBME‐1, and galectin‐3, with negativity for thyroglobulin; loss of CD56 expression was also noted.

The postoperative course was uneventful. Serum CEA declined from 20.8 to 3.1 ng/mL, and serum calcitonin declined from 2100 to 3.1 ng/L. At the 3‐month postoperative follow‐up, the patient remained in good general condition without active complaints.

## 5 Discussion

Medullary thyroid cancer (MTC) is a rare neuroendocrine neoplasm arising from parafollicular C cells that accounts for only 1%–2% of all thyroid malignancies [1, 2]; although most cases are sporadic, germline or somatic variants in the RET proto‐oncogene play a central role in disease pathogenesis. Taken together, the five studies discussed below make clear that the continuum from diagnosis to advanced‐stage treatment can no longer be approached through a single biomarker or a standardized surgical protocol, but instead requires a multilayered, individualized approach.

While the initial biochemical trigger for further evaluation in our patient was an isolated procalcitonin elevation identified during an unrelated metabolic work‐up, procalcitonin secretion is itself a recognized physiological product of thyroid parafollicular C cells, and its elevation, once a C‐cell neoplasm is present, is therefore an expected rather than a distinguishing biochemical feature [3]. The concurrently elevated CEA (20.8 ng/mL) is, in our view, the more diagnostically instructive finding. CEA is not a C‐cell‐specific analyte in the same intuitive sense as calcitonin, and an isolated CEA elevation in an older adult is conventionally pursued as a possible marker of gastrointestinal malignancy; several reports have specifically described MTC presenting with an initial, unexplained CEA elevation that was ultimately traced to the thyroid [4, 5]. Akbulut and Sogutcu reported a directly comparable presentation, a patient with subclinical hyperthyroidism and sporadic MTC first suspected because of an isolated high CEA level, with strong CEA immunoreactivity later confirmed on the surgical specimen [4]. In a larger retrospective series, preoperative CEA has also been shown to correlate with tumor size and stage, with concentrations above approximately 270–400 ng/mL associated with larger or metastatic disease [6]; the comparatively modest CEA elevation in our patient is concordant with the small, low‐grade, node‐negative (pN0) tumor ultimately identified on pathology. We also note that CEA interpretation may be confounded by chronic kidney disease, which can independently cause mild CEA elevation; nonetheless, the concurrent, proportionate elevation of calcitonin supported a thyroid rather than a renal or gastrointestinal origin.

Although calcitonin remains an indispensable biomarker in the diagnostic workup, its immunohistochemical specificity is not absolute. In the case reported by Stezzi et al., calcitonin positivity identified on biopsy of a lung nodule was initially interpreted as metastatic MTC; however, normal serum calcitonin, chromogranin A, and CEA levels, together with the absence of a RET mutation, ultimately established the lesion as a low‐grade pulmonary neuroendocrine tumor [7]. This case demonstrates that calcitonin staining in extrathyroidal tissue may fall well short of the near‐100% specificity observed in thyroid fine‐needle aspiration, underscoring the need to interpret histopathological findings in conjunction with the clinical and biochemical profile. In the same case, the failure of DOTATATE PET‐CT to detect a low‐grade tumor with weak somatostatin receptor expression further highlights the need to individualize radiotracer selection according to tumor biology.

A similar question arises regarding the prediction of distant metastatic risk at diagnosis. In a cohort of 81 patients, Schonebaum et al. [8] showed that the 500 pg/mL calcitonin threshold recommended by the current ATA guidelines has high sensitivity (90.9%) but low specificity (47.5%), such that more than half of M0 patients are exposed to unnecessary imaging. On multivariable analysis, the presence of a suspicious lymph node on ultrasonography was a stronger independent predictor than calcitonin level (OR 6.71 vs. 1.85), and a model combining both variables achieved an AUC of 0.85. The alternative algorithm proposed by the authors recommends imaging for all patients with a suspicious lymph node, as well as for those without a suspicious node whose calcitonin exceeds 180 pg/mL; this approach preserves sensitivity while substantially improving specificity. These findings suggest that imaging decisions would be more rationally based on a composite model incorporating lymph node status rather than on a single threshold value.

A similar need for individualization emerges when determining the extent of surgery. In the 389‐patient series reported by Cappagli et al. [9], multifocality was present in 14.5% of sporadic cases versus 56.4% of hereditary cases, and bilaterality in 8.7% and 56.4%, respectively. Neither preoperative variables such as age, sex, and tumor diameter, nor pathological features reliably predicted multifocality or bilaterality, and a substantial proportion of contralateral foci were missed on preoperative ultrasonography. Nonetheless, the low bilaterality rate observed in selected sporadic cases without a germline RET mutation or preoperative lateral lymph node involvement provides evidence that lobectomy may be a safe option in this subgroup, whereas total thyroidectomy remains indispensable in hereditary MTC because of its multiclonal genetic basis.

Regarding the choice of systemic therapy in advanced or metastatic disease, the network meta‐analysis by Wang et al. defined a clear hierarchy among RET‐targeted agents [10]. The selective RET inhibitor selpercatinib achieved a 90% reduction in the risk of progression relative to placebo (HR 0.10) and a superior objective response rate (OR 122.6), while largely avoiding the off‐target toxicities—such as hypertension, diarrhea, and QTc prolongation—that are common with multikinase inhibitors such as cabozantinib and vandetanib; a hepatotoxicity signal (OR 4.20) remained a notable exception requiring clinical monitoring. The authors noted that this advantage is most strongly supported in first‐line, RET‐mutant patients, and that caution is warranted when generalizing these findings to other populations. On the other hand, acquired resistance mediated by RET solvent‐front mutations (G810R/S/C) has emerged as an important clinical challenge that may limit the long‐term efficacy of selective inhibitors.

Finally, the pioneering study by Jager et al. [11] demonstrated that patient‐derived MTC organoids closely recapitulate the original tumor tissue with respect to gene–protein expression, calcitonin–CEA secretion, and response to tyrosine kinase inhibitors. The organoids' preserved self‐renewal capacity across serial passages and their faithful retention of the original tumor's RET mutation support the model's genetic reliability as a representative platform. The same drug panel produced varying degrees of calcitonin–CEA suppression across different patients, and the organoids took up 18F‐FDG and 18F‐DOPA but failed to respond specifically to 18F‐PSMA because of a lack of vascularization—findings that suggest this platform could guide personalized drug and radiotracer selection in the future, although multicenter validation will be required before clinical use.

This case has notable strengths, including a comprehensive biochemical work‐up (calcitonin, CEA, and procalcitonin), a full immunocytochemical and immunohistochemical panel confirming the diagnosis, and biochemical follow‐up demonstrating a clear postoperative response.

## 6 Limitations

This report has several limitations. Germline RET mutation testing could not be performed, precluding genotype‐based risk stratification and formal exclusion of an occult hereditary syndrome. Representative photomicrographs of CEA and procalcitonin immunohistochemical staining could not be included, as the original stained slides were not retained by the pathology laboratory and are therefore no longer available for re‐imaging. Finally, as a single‐patient report, this case cannot establish how generalizable procalcitonin elevation is as a diagnostic clue in other patients with concurrent chronic kidney disease.

In summary, these five studies together highlight the importance of recognizing the pitfalls of calcitonin immunostaining in the management of MTC, incorporating lymph node status into imaging decisions, considering more conservative surgery in selected sporadic cases, guiding treatment selection by RET mutation status in advanced disease, and the emerging future role of organoid‐based functional testing. However, because most of these findings derive from retrospective, single‐center studies, prospective, multicenter validation is needed before substantial changes to clinical guidelines can be recommended. In clinical practice, the current evidence supports decision‐making within multidisciplinary tumor boards that evaluate each patient's biochemical profile, imaging findings, and genetic status as an integrated whole.

This case demonstrates that medullary thyroid carcinoma can remain entirely silent until an incidental biochemical abnormality—here, an isolated procalcitonin elevation rather than the expected calcitonin trigger—prompts further evaluation. In patients with chronic kidney disease, calcitonin elevation should not be reflexively attributed to reduced renal clearance; excluding an underlying C‐cell malignancy remains essential. Greater awareness of this atypical presentation may support earlier diagnosis and better outcomes in similarly asymptomatic patients.

## Author Contributions

**Orhan Altınyüzük:** investigation, writing – original draft, writing – review and editing, resources, formal analysis. **Bartu Ediz:** investigation. **Serkan Feyyaz Yalın:** investigation, writing – review and editing. **Ergün Parmaksız:** conceptualization, investigation, writing – original draft, validation.

## Funding

The authors have nothing to report.

## Ethics Statement

Ethical approval was not required for this case report, as no experimental interventions were performed and all patient information was anonymized in accordance with institutional and international guidelines.

## Consent

Written informed consent for publication of this case report, including clinical details and any accompanying data, was obtained from the patient.

## Conflicts of Interest

The authors declare no conflicts of interest.

## Data Availability

The datasets generated during and/or analyzed during the current study are available from the corresponding author upon reasonable request.
